# Pan-coronavirus fusion inhibitors as the hope for today and tomorrow

**DOI:** 10.1007/s13238-020-00806-7

**Published:** 2021-01-09

**Authors:** Xinling Wang, Shuai Xia, Yun Zhu, Lu Lu, Shibo Jiang

**Affiliations:** 1 Key Laboratory of Medical Molecular Virology (MOE/NHC/CAMS), School of Basic Medical Sciences, Fudan University, Shanghai, 200032 China; 2 National Laboratory of Biomacromolecules, Institute of Biophysics, Chinese Academy of Sciences, Beijing, 100101 China

The last two decades of the 21st century have seen emerging zoonotic coronavirus (CoV) diseases, including severe acute respiratory syndrome (SARS) (Holmes 2003), Middle East respiratory syndrome (MERS) (Graham et al., 2013) and coronavirus disease 2019 (COVID-19) (Jiang et al., 2020), all posing a devastating threat to global public health and economy. Human common coronavirus 229E (HCoV-229E), HCoV-NL63 and HCoV-OC43 can cause upper respiratory infection in adults and children, even leading to fatal diseases (Morfopoulou et al., 2016; Konca et al., 2017; Veiga et al., 2020). Moreover, recent studies suggested that some bat-derived SARS-related coronaviruses (SARSr-CoVs) have the potential to cause new CoV diseases in the future (Cui et al., 2019). This calls for the development of pan-CoV inhibitors to combat both current and future pandemics or epidemics of CoV diseases.

## Conserved target site in coronavirus S protein

**Figure 1:**
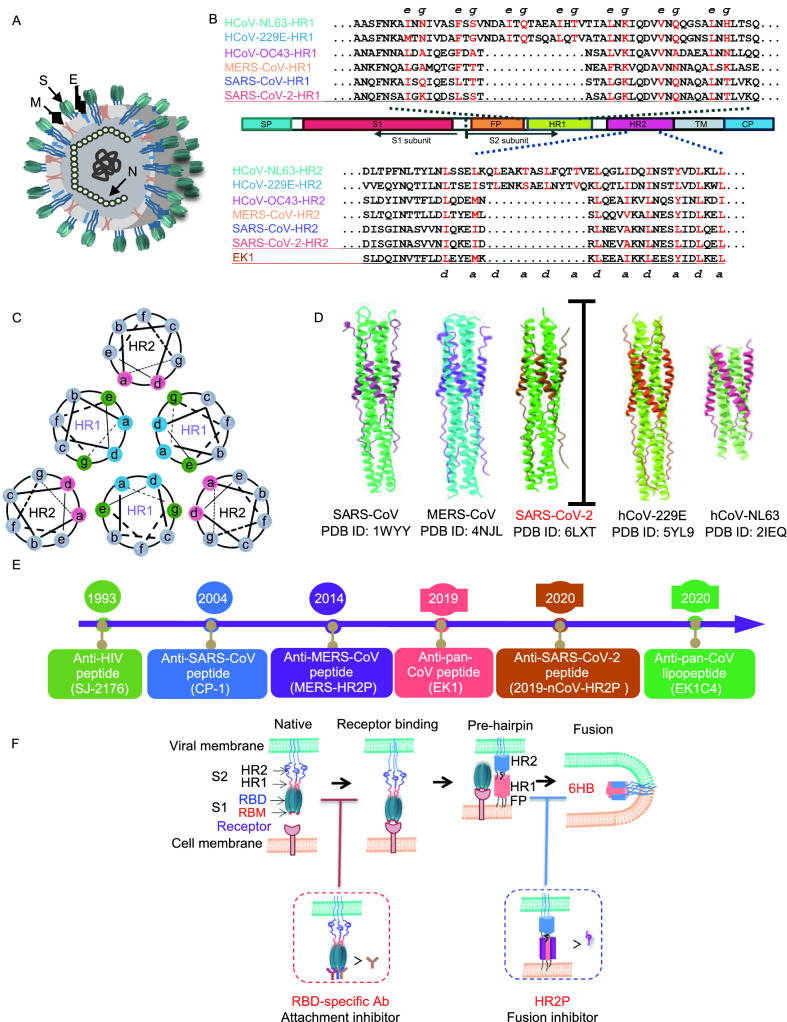
Research and development of peptide-based virus fusion inhibitors. (A) The structural protein of coronavirus. There are three transmembrane proteins, including spike protein (S; celadon), membrane protein (M; orange), and envelope protein (E; blue) on the surface of envelope, in addition to a nucleocapsid protein (N; cyan) inside the virion. (B) Alignment of the conserved HR1 and HR2 sequences of human CoVs. Spike (S) protein consists of signal peptide (SP), receptor-binding domain (RBD), fusion peptide (FP), heptad repeat 1 domain (HR1), heptad repeat 2 domain (HR2), transmembrane domain (TM), and cytoplasmic domain (CP). The amino acid sequence of EK1 is also shown in the figure. (C) The model of 6-HB formation between HR1 and HR2 in S2 subunit of human CoV S protein. In the 6-HB formation process, three HR1 helices form inter trimer by the interaction of residues at “a” and “d” position (shown as the blue ball), resulting in the exposure of three hydrophobic grooves where HR2 helices will bind. Then, the residues at “e” and “g” position (shown as the green ball) in HR1 helices interact with the residues at “a” and “d” position (shown as the pink ball) in HR2 helices to form 6-HB structure. (D) The crystal structure of 6-HB formed by HR1 and HR2 domains of different human CoVs. (E) Milestones of the peptide-based virus fusion inhibitors. (F) The mechanism of human CoV S protein-mediated virus attachment and fusion and the mechanism of action of the attachment and fusion inhibitors. In the native state, the S2 subunit is encapsulated in the S1 subunit. After receptor engagement by viral RBD, several conformation changes occur in the S2 subunit. Three HR1 molecules form HR1-trimer core structure, and three HR2 molecules interact with HR1-trimer to form 6-HB, mediating membrane fusion. An RBD-specific neutralizing antibody inhibits viral infection by blocking the binding of RBD to the cellular receptor. A fusion inhibitor inhibits the membrane fusion process by blocking 6-HB formation

The development of pan-CoV inhibitors depends on identifying a conserved target site. As an enveloped virus, human coronavirus (HCoV) presents a spike protein (S) on the viral membrane surface. S protein plays key roles in virus entry, including receptor recognition, binding, and membrane fusion (Fig. 1A). It is a class I transmembrane glycoprotein, which includes two subunits, S1 and S2. S2 subunit consists of fusion peptide (FP), heptad repeat 1 and 2 domains (HR1 and HR2), transmembrane domain (TM), and cytoplasmic domain (CP) (Fig. 1B). After binding between the receptor binding domain (RBD) in S1 subunit and a cellular receptor, a series of conformation changes in the S2 subunit are triggered. FP is exposed and inserts into the cell membrane. Then, three HR1 domains associate with each other to form an internal trimer with three exposed hydrophobic grooves. The internal trimer is maintained by the interaction between residues located at the “a“ and “d“ positions in HR1 helices, and their “e”, “g” positions are exposed and interact with “a”, “d” positions in the HR2 helices to form a six-helix bundle (6-HB) (Fig. 1C), which brings the viral and target cell membranes into close proximity for fusion. The 6-HB formation is a conserved and critical mechanism for viral fusion and entry, and it is shared by all coronaviruses, mainly mediated by HR1 and HR2 regions. Sequence analysis, however, discovered that two α-coronaviruses (HCoV-229E and HCoV-NL63) have 14 amino acid insertions in both HR1 and HR2 regions comparable to those of β-coronavirus (HCoV-OC43, MERS-CoV, SARS-CoV, and SARS-CoV-2) (Fig. 1B). Crystal structure analysis of HR1-HR2 complexes has consistently indicated that the HR1-HR2 of those HCoVs showed similar 6-HB structures, but that the 6-HB of HCoV-229E and HCoV-NL63 exhibited much longer and bending helix in the HR2 domain (Xia et al., 2020b) (Fig. 1D).

## Anti-coronavirus peptide fusion inhibitors

Similar to the first HIV-1 fusion inhibitory peptide, SJ-2176, derived from the HIV-1 gp41 HR2 domain (Jiang et al., 1993), numerous HCoV fusion inhibitory peptides derived from their S protein HR2 domains have been identified (Fig. 1E), such as SARS-CoV fusion inhibitory peptide, CP-1 (Liu et al., 2004), MERS-CoV fusion inhibitory peptide, MERS-HR2P (Lu et al., 2014), and SARS-CoV-2 fusion inhibitory peptide, 2019-nCoV-HR2P (Xia et al., 2020a). Nevertheless, most of these HR2-derived fusion inhibitors showed little, or no, cross inhibitory activity against infection of heterologous HCoV, mainly because of the sequence difference in HR1 regions of these HCoVs. Sequence analysis of HR2 region showed that some residues belong to MERS-CoV are different from SARS-CoV and SARS-CoV-2. MERS-CoV has residues of M16, V22, Y29 at “a” position and L12 at “d” position, while these residues of SARS-CoV and SARS-CoV-2 are I12, I16, A22 and L29 (Fig. 1B). These differences may affect its interaction with the HR1 region of other CoVs to form the 6-HB core region, causing low inhibitory activity, which limits the broad-spectrum anti-CoVs activity. The secondary structure and chemical modification of peptide may also affect its anti-CoVs activity.

## The development of pan-Cov fusion inhibitors and their antiviral mechanism

After a series of screening and optimization, we found a peptide derived from the HR2 domain of HCoV-OC43, designated OC43-HR2P, showing broad fusion inhibitory activity against all five pseudotyped human coronaviruses (HCoVs) tested, including HCoV-229E, HCoV-NL63, HCoV-OC43, SARS-CoV and MERS-CoV. We then modified OC43-HR2P by introducing negatively and positively charged amino acids Glu (E) and Lys (K), respectively, at the i to i + 3 or i to i + 4 positions in a helix, to allow them to form intramolecular E–K or K–E salt bridges. The resultant optimized peptide EK1 has shown significantly improved fusion inhibitory activity against the above 5 HCoVs. EK1 also potently inhibits cell-cell fusion mediated by S proteins of the SARS-CoV-2 and 3 SARSr-CoVs (Rs3367, WIV1, SHC014) tested (Xia et al., 2019, 2020b). Furthermore, it is effective in inhibiting infection of above 6 pseudotyped HCoVs and SARSr-CoVs (Rs3367, WIV1), as well as five live HCoVs (HCoV-229E, HCoV-NL63, HCoV-OC43, SARS-CoV-2, and MERS-CoV) (Xia et al., 2020b).

Based on the crystal structures, we found that EK1 properly fits into the hydrophobic groove formed by two HR1 helices and that the binding site is consistent with those of native HR2s, forming a similar 6-HB structure. Some hydrophobic residues of EK1, such as L12, M16, L19, I23, L26, as shown in red font in Fig. 1B, formed direct and extensive hydrophobic interactions with HR1, while some residues with long side chains, such as E15, K18, E27, and Y30, also formed reliable hydrophilic interactions with HR1. Moreover, the hydrophobic residues of EK1, e.g., L36, can insert deeply into hydrophobic pockets formed by adjacent HR1s, promoting the terminal region of EK1 to adhere to 3HR1 cores. Interestingly, the residues on HR1 mediating the hydrophobic interaction with EK1 are conserved across all HCoVs, including both α-HCoV and β-HCoV. Meanwhile, those studies also confirmed that HCoV-HR2 regions could serve as conserved targeted sites for the development of broad-spectrum antiviral agents against multiple HCoV infections (Xia et al., 2019).

We then designed and synthesized a lipopeptide, EK1C4, by conjugating a cholesterol to the C-terminus of EK1 in order to improve the antiviral activity and half-life of EK1. Previous study has shown that conjugation with cholesterol is an effective strategy to enhance activity of HIV-1 fusion/entry inhibitors (Hollmann et al., 2013), possibly because the cholesterol group in the lipopeptide anchors to the target membrane or binds to the hydrophobic groove on HR1-trimer (Xia et al., 2020b). Surprisingly, the inhibitory activity of EK1C4 is about 241- and 149-fold more potent than EK1 against SARS-CoV-2 S protein-mediated membrane fusion (IC_50_: 1.3 nmol/L vs. 315 nmol/L) and pseudovirus infection (IC_50_: 15.8 nmol/L vs. 2,375 nmol/L), respectively. Its inhibitory activity against live SARS-CoV-2 infection (IC_50_: 36.5 nmol/L) is about 30-fold and 20-fold more potent than chloroquine (IC_50_: 1,130 nmol/L) and remdesivir (IC_50_: 770 nmol/L), respectively (Wang et al., 2020). EK1C4 also exhibits highly improved inhibitory activity against all the above HCoVs and SARSr-CoVs tested, suggesting that EK1C4 has the potential to be developed as a pan-coronavirus prophylatic or therapeutic to prevent or treat infection by the current SARS-CoV-2 and other emerging and reemerging SARSr-CoV and SARS-CoV (Xia et al., 2020b).

## COMBINATORIAL USE OF A PAN-COV FUSION INHIBITOR WITH A POTENT NEUTRALIZING ANTIBODY TARGETING RBD IN S PROTEIN OF A CORONAVIRUS

A potent neutralizing antibody targeting the RBD in S protein of SARS-CoV-2 has the potential to prevent and treat COVID-19 (Wu et al., 2020). However, RBD is a highly mutable region in the coronavirus S protein. Indeed, numerous studies have shown that a large number of escape mutations are enriched in HCoV RBD (Wong et al., 2017; Kleine-Weber et al., 2019; Ou et al., 2020). Therefore, the combinatorial use of an RBD-specific neutralizing antibody and a pan-CoV fusion inhibitor is expected to be effective against a coronavirus with mutations in RBD. It may also show potent synergistic antiviral effect against divergent HCoVs. For example, combining the fusion peptide HR2P-M2 derived from the MERS-CoV HR2 domain and RBD-specific neutralizing antibody m336 showed synergism against MERS-CoV with or without mutations in the RBD region (Wang et al., 2019). Therefore, combining a pan-CoV fusion inhibitor, EK1 or EK1C4, with an RBD-specific neutralizing antibody (Fig. 1F) can combat pandemics or epidemics of COVID-19, SARS, MERS or the emerging and reemerging coronavirus diseases that may be caused by a SARSr-CoV in the future.

## Prospects for future

In the last two decades, we have seen the continuous emergence of novel highly pathogenic HCoVs to seriously threaten global public health and economy, while some animal coronaviruses, such as bat-SARSr-CoVs, still have the potential to cross the species-barrier to infect humans in the future. This reality calls for the development of potent and broad-spectrum antiviral agents against current, as well as the emerging and reemerging, HCoVs. We have identified highly potent pan-CoV fusion inhibitors, EK1 and EK1C4, which target the conserved HR1 region in S protein of HCoVs, with several advantages, including low immunogenicity, good safety and druggability, particularly useful for short-term application in the early stage of coronavirus infection to save patients’ lives (Fosgerau and Hoffmann 2015; Xia et al., 2019, 2020b). However, this target is not good for developing pan-CoV neutralizing antibodies because: 1) the HR1-trimer has low immunogenicity since it is only instantly exposed to immune system during the membrane fusion stage, and 2) becuase of the steric hindrance, the antibody IgG (~150 kDa) is too big to access to the transitly exposed HR1-trimer, which is accessible to a molecule ranging from 6 to 41 kDa (Hamburger et al., 2005). Since a single-domain antibody, e.g., a nanobody, with a molecular weight of ~15 kDa (Wu et al., 2020) can access to the HR1-trimer, it is possible to develop pan-CoV neutralizing nanobodies targeting the HR1-trimer of coronaviruses. Another disadvantage of a peptide drug is its relative short half-life. Therefore, it is still necessary to optimize the pan-CoV fusion inhibitor to further improve its antiviral potency and half-life. Our previous study has shown that conjugation of an IgG Fc-binding motif to an HIV-1 fusion inhibitory peptide can significantly improve the peptide’s half-life (Bi et al., 2019; Xia et al., 2020b). The strategy of designing cyclic peptides (Nielsen et al., 2017) may further improve stability, even making oral administration available. The combination of a pan-CoV fusion inhibitor with a potent RBD-specific neutralizing antibody with strong synergism is expect to reduce the dosage of antibody and peptide used, thus reducing the cost to patients, in addition to the benefits of combination (or cocktail) therapy mentioned above. Overall, the pan-CoV fusion inhibitors show promise for further development to combat present and future coronavirus pandemics.

## References

[CR1] Bi W, Xu W, Cheng L, Xue J, Wang Q, Yu F, Xia S, Wang Q, Li G, Qin C (2019). IgG Fc-binding motif-conjugated HIV-1 fusion inhibitor exhibits improved potency and in vivo half-life: Potential application in combination with broad neutralizing antibodies. PLoS Pathog.

[CR2] Cui J, Li F, Shi ZL (2019). Origin and evolution of pathogenic coronaviruses. Nature Reviews Microbiology.

[CR3] Fosgerau K, Hoffmann T (2015). Peptide therapeutics: current status and future directions. Drug Discov Today.

[CR4] Graham RL, Donaldson EF, Baric RS (2013). A decade after SARS: strategies for controlling emerging coronaviruses. Nature Reviews Microbiology.

[CR5] Hamburger AE, Kim S, Welch BD, Kay MS (2005). Steric accessibility of the HIV-1 gp41 N-trimer region. J Biol Chem.

[CR6] Hollmann A, Matos PM, Augusto MT, Castanho MARB, Santos NC (2013). Conjugation of cholesterol to HIV-1 fusion inhibitor C34 increases peptide-membrane interactions potentiating its action. Plos ONE.

[CR7] Holmes KV (2003). SARS coronavirus: a new challenge for prevention and therapy. J Clin Invest.

[CR8] Jiang S, Du L, Shi Z (2020). An emerging coronavirus causing pneumonia outbreak in Wuhan, China: calling for developing therapeutic and prophylactic strategies. Emerg Microbes Infect.

[CR9] Jiang S, Lin K, Strick N, dlx AR (1993). Hiv-1 inhibition by a peptide. Nature.

[CR10] Kleine-Weber H, Elzayat MT, Wang LS, Graham BS, Muller MA, Drosten C, Pohlmann S, Hoffmann M (2019). Mutations in the Spike Protein of Middle East respiratory syndrome coronavirus transmitted in Korea increase resistance to antibody-mediated neutralization. J Virol.

[CR11] Konca C, Korukluoglu G, Tekin M, Almis H, Bucak IH, Uygun H, Altas AB, Bayrakdar F (2017). The First Infant Death Associated with Human Coronavirus Nl63 Infection. Pediatr Infect Dis J.

[CR12] Liu S, Xiao G, Chen Y, He Y, Niu J, Escalante CR, Xiong H, Farmar J, Debnath AK, Tien P (2004). Interaction between heptad repeat 1 and 2 regions in spike protein of SARS-associated coronavirus: implications for virus fusogenic mechanism and identification of fusion inhibitors. Lancet.

[CR13] Lu L, Liu Q, Zhu Y, Chan KH, Qin L, Li Y, Wang Q, Chan JF, Du L, Yu F (2014). Structure-based discovery of Middle East respiratory syndrome coronavirus fusion inhibitor. Nat Commun.

[CR14] Morfopoulou S, Brown JR, Davies EG, Anderson G, Virasami A, Qasim W, Chong WK, Hubank M, Plagnol V, Desforges M (2016). Human coronavirus OC43 associated with fatal encephalitis. New Engl J Med.

[CR15] Nielsen DS, Shepherd NE, Xu WJ, Lucke AJ, Stoermer MJ, Fairlie DP (2017). Orally absorbed cyclic peptides. Chem Rev.

[CR16] Ou J, Zhou Z, Dai R, Zhang J, Lan W, Zhao S, Wu J, Donald Seto, Cui L, Zhang G (2020). Emergence of RBD mutations in circulating SARS-CoV-2 strains enhancing the structural stability and human ACE2 receptor affinity of the spike protein. Preprint at.

[CR17] Veiga A, Martins LG, Riediger I, Mazetto A, Debur MDC, Gregianini TS (2020). More than just a common cold: Endemic coronaviruses OC43, HKU1, NL63, and 229E associated with severe acute respiratory infection and fatality cases among healthy adults. J Med Virol.

[CR18] Wang C, Hua C, Xia S, Li W, Lu L, Jiang S (2019). Combining a fusion inhibitory peptide targeting the MERS-CoV S2 protein HR1 domain and a neutralizing antibody specific for the S1 protein receptor-binding domain (RBD) showed potent synergism against pseudotyped MERS-CoV with or without mutations in RBD. Viruses-Basel.

[CR19] Wang M, Cao R, Zhang L, Yang X, Liu J, Xu M, Shi Z, Hu Z, Zhong W, Xiao G (2020). Remdesivir and chloroquine effectively inhibit the recently emerged novel coronavirus (2019-nCoV) in vitro. Cell Res.

[CR20] Wong AHM, Tomlinson ACA, Zhou DX, Satkunarajah M, Chen K, Sharon C, Desforges M, Talbot PJ, Rini JM (2017). Receptor-binding loops in alphacoronavirus adaptation and evolution. Nat Commun.

[CR21] Wu Y, Li C, Xia S, Tian X, Kong Y, Wang Z, Gu C, Zhang R, Tu C, Xie Y (2020). Identification of human single-domain antibodies against SARS-CoV-2. Cell Host Microbe.

[CR22] Xia S, Zhu Y, Liu M, Lan Q, Xu W, Wu Y, Ying T, Liu S, Shi Z, Jiang S (2020). Fusion mechanism of 2019-nCoV and fusion inhibitors targeting HR1 domain in spike protein. Cell Mol Immunol.

[CR23] Xia S, Liu M, Wang C, Xu W, Lan Q, Feng S, Qi F, Bao L, Du L, Liu S (2020). Inhibition of SARS-CoV-2 (previously 2019-nCoV) infection by a highly potent pan-coronavirus fusion inhibitor targeting its spike protein that harbors a high capacity to mediate membrane fusion. Cell Res.

[CR24] Xia S, Yan L, Xu W, Agrawal AS, Algaissi A, Tseng CK, Wang Q, Du L, Tan W, Wilson IA (2019). A pan-coronavirus fusion inhibitor targeting the HR1 domain of human coronavirus spike. Sci Adv.

